# Comparison of voltages between atria: differences in sinus rhythm and atrial fibrillation

**DOI:** 10.1007/s10840-023-01671-0

**Published:** 2023-10-24

**Authors:** Alexander P. Bates, John Paisey, Arthur Yue, Phil Banks, Paul R. Roberts, Waqas Ullah

**Affiliations:** 1 Department of Cardiology, University Hospital Southampton NHS Foundation Trust, Tremona Road, Southampton, UK; 2 Faculty of Medicine, School of Human Development and Health, University of Southampton, Southampton, UK

**Keywords:** Mapping, Atrial fibrillation, Atrial fibrosis, Low voltage areas

## Abstract

**Background:**

Ultra high-density mapping systems allow for comparison of atrial electroanatomical maps in unprecedented detail. Atrial scar determined by voltages and surface area between atria, rhythm and atrial fibrillation (AF) types was assessed.

**Methods:**

Left (LA) and right atrial (RA) maps were created using Rhythmia HDx in patients listed for ablation for paroxysmal (PAF, sinus rhythm (SR) maps only) or persistent AF (PeAF, AF and SR maps). Electrograms on corresponding SR/AF maps were paired for direct comparison. Percentage surface area of scar was assigned low- (LVM, ≤ 0.05 mV), intermediate- (IVM, 0.05–0.5 mV) or normal voltage myocardium, (NVM, > 0.5 mV).

**Results:**

Thirty-eight patients were recruited generating 96 maps using 913,480 electrograms. Paired SR-AF bipolar electrograms showed fair correlation in LA (Spearman’s *ρ* = 0.32) and weak correlation in RA (*ρ* = 0.19) and were significantly higher in SR in both (LA: 0.61 mV (0.20–1.67) vs 0.31 mV (0.10–0.74), RA: 0.68 mV (0.19–1.88) vs 0.47 mV (0.14–1.07), *p* < 0.0005 both). Voltages were significantly higher in patients with PAF over PeAF, (LA: 1.13 mV (0.39–2.93) vs 0.52 mV (0.16–1.49); RA: 0.93 mV (0.24–2.46) vs 0.57 mV (0.17–1.69)). Minimal differences were seen in electrogram voltages between atria.

Significantly more IVM/LVM surface areas were seen in AF over SR (LA only, *p* < 0005), and PeAF over PAF (LA: *p* = 0.01, RA: *p* = 0.04). There was minimal difference between atria within patients.

**Conclusions:**

Ultra high-density mapping shows paired electrograms correlate poorly between SR and AF. SR electrograms are typically (but not always) larger than those in AF. Patients with PeAF have a lower global electrogram voltage than those with PAF. Electrogram voltages are similar between atria within individual patients.

**Supplementary information:**

The online version contains supplementary material available at 10.1007/s10840-023-01671-0.

## Introduction

Atrial fibrillation (AF) is an arrhythmia associated with progressive mural fibrosis [1]. Atrial fibrosis has been shown to harbour electrical triggers for AF [2], and when represented as low voltage areas on 3D electroanatomical maps (EAM), it serves as a target for ablation. Consequently, accurate representation and understanding of atrial substrate on 3D electroanatomical maps are vital, particularly as scar-guided ablation strategies beyond pulmonary vein isolation (PVI) are showing promise [3].

Atrial fibrosis is depicted on 3D EAMs using bipolar voltage as a surrogate. Studies have investigated the relationships of bipolar voltage with AF progression [2, 4, 5] and between corresponding SR and AF electrograms [6–9]. These studies required selection and review of electrograms on a manual basis. However, the advent of new ultrahigh-density mapping systems allows for the swift acquisition of thousands of electrograms which can be verified on an automated basis. Consequently, analyses can now be undertaken at an unprecedented level of detail.

In this study, an ultrahigh-density mapping system was used to investigate the relationships of electrogram voltage between SR and AF, patients with PAF versus PeAF and finally between left and right atria.

## Materials and methods

### Patient selection

Patients listed for radiofrequency AF ablation at our centre were prospectively recruited as part of the ‘High Density Scar Guided Atrial Fibrillation Mapping’ (HD-SAGA, NCT03363087) study. Ethical approval was granted by the UK Research and Ethics Committee, (Reference: 18/SC/0077). Informed written consent was taken from all study participants.

All persistent AF patients were in AF at the time of their procedure.

### Procedure

Procedures were performed under general anaesthetic or local anaesthetic with conscious sedation. A decapolar catheter was placed into the coronary sinus as a reference for the creation of 3D electroanatomical maps. Voltage data was collected using the INTELLAMAP Orion catheter with the RHYTHMIA HDx system, (Boston Scientific, Marlborough, MA, USA).

Additional data collection required in a separate part of the HD-SAGA study meant the mapping protocol was adjusted during recruitment. This was due to procedural time constraints. For the first 19 patients recruited, mapping of both atria occurred whilst the second set only underwent mapping of the LA. The systematic mapping protocol is summarised in Supplementary Materials Fig. 1. The reason for only mapping the LA for the second set of 19 patients was due to studying other electrophysiological relationships in the left atrium as part of the HD-SAGA protocol. Consequently, due to procedural time constraints, the RA was not mapped in these cases. Maps in SR were collected during proximal coronary sinus pacing.

Mapping points were acquired ensuring all areas of the 3D anatomical shell had bipolar voltage data ascribed, using the automated acceptance criteria of the system. For this purpose, the colour fill threshold was set at 5.0 mm and confidence mask of 0.03 mV. Electrogram voltages were classified as normal- (NVM, > 0.5 mV), intermediate- (IVM, 0.05–0.5 mV) or low voltage myocardium (LVM, ≤ 0.05 mV). In areas where no colour was ascribed, minimal electrical activity was confirmed by real-time manual review of electrograms on the Orion catheter. For AF, accepted electrograms were respiratory gated, with catheter motion < 1.0 mm. For SR, accepted electrograms additionally showed timing stability to the proximal coronary sinus reference electrogram, and a cycle length stability, (both < 5.0 ms).

### Data collection and analysis

Post-procedure, to ensure only data from the atria were analysed, sites such as the pulmonary veins, vena cavae and value apparatus were excluded from analysis using the Rhythmia cut-out tool. Mapping data were exported from Rhythmia and analysed using custom MATLAB scripts (Mathworks Inc., Natick, MA, USA). Electrogram voltages were co-located with their respective map xyz co-ordinates on the export.

To examine the relationship of voltage amplitude between SR and AF, electrograms on corresponding maps were paired with their nearest counterpart based on their xyz co-ordinates. An electrogram could only be paired once to avoid repeat comparisons. Electrograms without a partner within 2.5 mm were excluded, a value based upon the distance between Orion electrodes. To examine the electrogram voltage differences between patients with PAF and PeAF, the global median voltage for each map was calculated and compared between groups. To examine for differences in electrogram voltages between atria, comparisons were made within patients.

To determine the percentage surface area attributed to LVM/IVM/NVM, the mapped area ascribed to each electrogram was calculated. The export connects every mapped electrogram to two neighbours, forming a triangle. Combining these triangles allows for the entire map to be reconstructed. From the xyz co-ordinates of each set of 3 electrograms, the area of a triangle can be calculated. Each triangle was then divided into three equal sections, each being assigned LVM / IVM / NVM based upon the voltages at its vertices.

### Statistical analysis

IBM SPSS Statistics (Version 27, IBM Corp, NY, USA) was used for statistical analysis. A *p*-value of < 0.05 was considered statistically significant. Variables were assessed as parametric or non-parametric by visual inspection of histograms and a Shapiro-Wilks test. Continuous data were expressed as mean ± SD or median (lower quartile, upper quartile). Count data were expressed as number (%). Bivariate correlations were performed using Pearson’s product moment correlation. Repeated data was analysed using a Wilcoxon signed rank test. Agreement of assignment to categories was assessed by Cohen’s Kappa. Linear regression was performed to assess modelling of continuous data. Independent samples were compared using Mann–Whitney U Test for medians. A generalized linear mixed model was used to compare data between atria and within patients (random factor). Comparison of data with multiple dependent variables was performed using a one-way MANOVA where data was independent and a repeated measures MANOVA where it was not.

## Results

### Patient and mapping characteristics

Thirty-eight patients were recruited to the study generating 96 maps (LA-SR 38, LA-AF 29, RA-SR 19, RA-AF 10) using a total of 913,480 electrograms. Patient details are described in Table 1. At the conclusion of each procedure, PVI had been successfully achieved and SR was maintained.

**Table 1:** Study population characteristics

Patient characteristics	
*n*	38
Female	18 (47.4%)
Age, years	67.2 ± 8.8
Body mass index (kg/m^2^)	31.1 ± 5.1
Co-morbidities	
Arterial hypertension	18 (47.4%)
Ischaemic heart disease	6 (15.8%)
Diabetes mellitus	3 (7.9%)
Stroke	2 (5.3%)
Heart failure	9 (23.7%)
COPD	1 (2.6%)
LVEF (%)	57.1 ± 8.2
CHA_2_DS_2_-VASc	2.4 ± 1.5
Type of atrial fibrillation	
Paroxysmal	9 (23.7%)
Persistent	8 (21.1%)
Long standing persistent	21 (55.3%)
Electrograms acquired	
Left atrium – SR	10,097 ± 2779
Left atrium – AF	10,296 ± 2176
Right atrium – SR	8119 ± 1937
Right atrium – AF	8290 ± 1802

Displayed as *n*, (%) or mean ± standard deviation. *AF* atrial fibrillation, *LVEF* left ventricular ejection fraction, *SR* sinus rhythm

### Sinus rhythm vs atrial fibrillation

There were moderate correlations between paired SR and AF electrogram voltages in the LA, (Pearson’s *r* – 0.32, *p* < 0.0005), but weak correlations in the RA, (*r* – 0.19, *p* < 0.0005). Linear regression showed LA-SR bipolar voltages could be statistically significantly predicted by their paired AF voltages, but the predictive ability was low (Adjusted R^2^ = 0.11, *p* < 0.0005). Similarly, for classifying voltages into LVM, IVM or NVM, there was only fair agreement between LA-SR and LA-AF (Cohen’s *κ* = 0.24, *p* < 0.0005). Bipolar RA-SR and paired RA-AF voltages showed weaker results (Adjusted R^2^ = 0.04, Cohen’s *κ* = 0.18, *p* < 0.0005 both).

Paired electrograms were significantly larger in SR than AF in both atria, (*p* < 0.0005, Fig. 2 and Supplementary Table 1). In the LA, the percentage surface areas denoted as LVM/IVM/NVM were significantly different between SR and AF, with AF having greater IVM and LVM than SR, (*p* < 0.0005). In the RA, a trend in this direction was seen but did not reach statistical significance, (*p* = 0.62, Fig. 3 and Supplementary Table 1).

Despite these results, a considerable proportion (29.5%) of the paired electrograms had a larger amplitude in AF than SR. Additionally, 11.4% of electrogram pairs had the AF electrogram placed in a healthier category than their SR counterpart, (for example, AF electrogram graded as NVM, whilst SR graded as IVM or LVM).

**Fig. 1:**
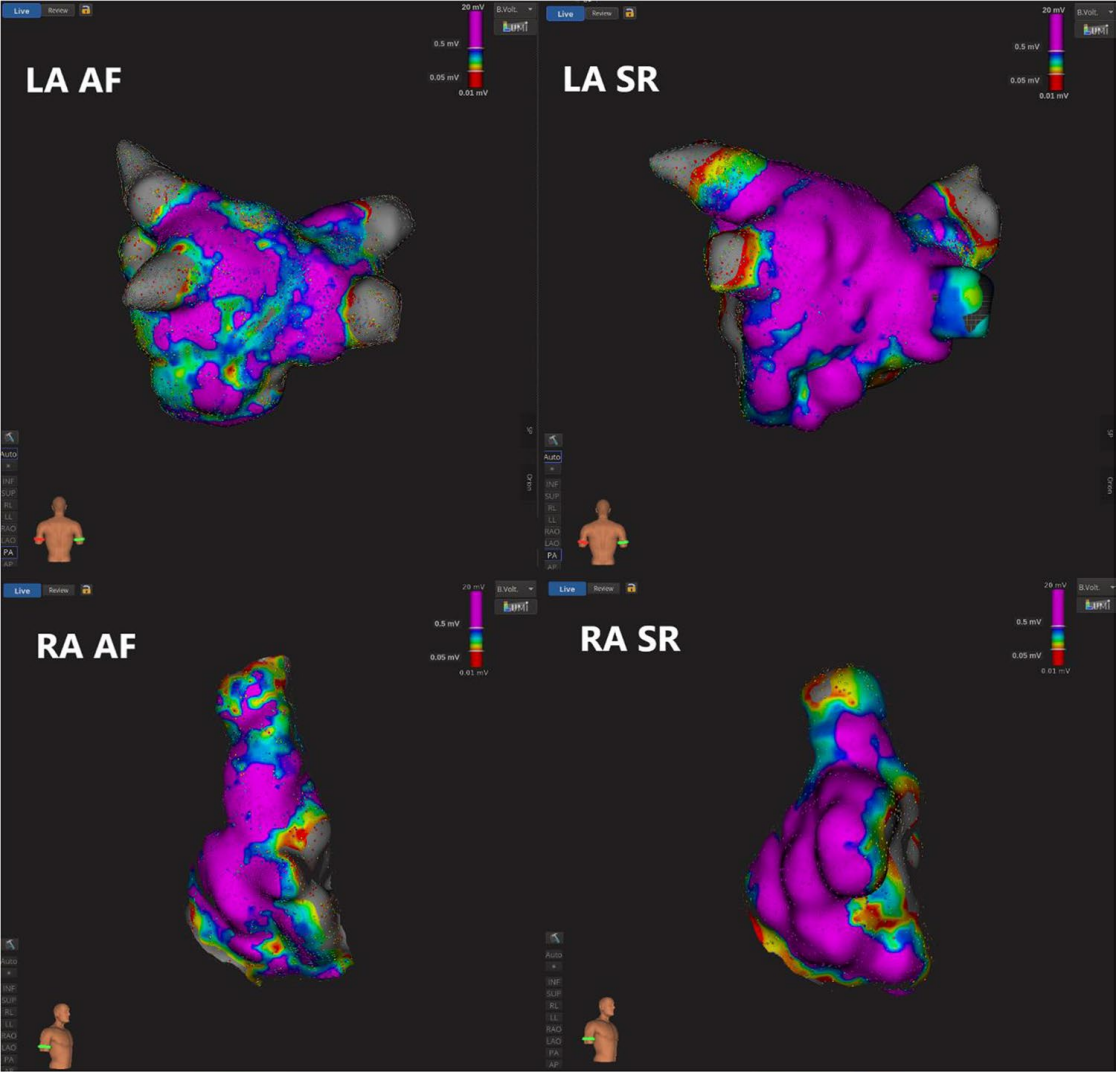
An example of 3D electroanatomical maps of the left (LA) and right (RA) atria between sinus rhythm (SR) and atrial fibrillation (AF) within the same patient

Typical examples of paired SR and AF maps are displayed in Fig. 1.

### Paroxysmal vs persistent atrial fibrillation

To compare parameters between patients with PAF or PeAF, maps created in SR were used, and a global median voltage for each map was calculated.

**Fig. 2:**
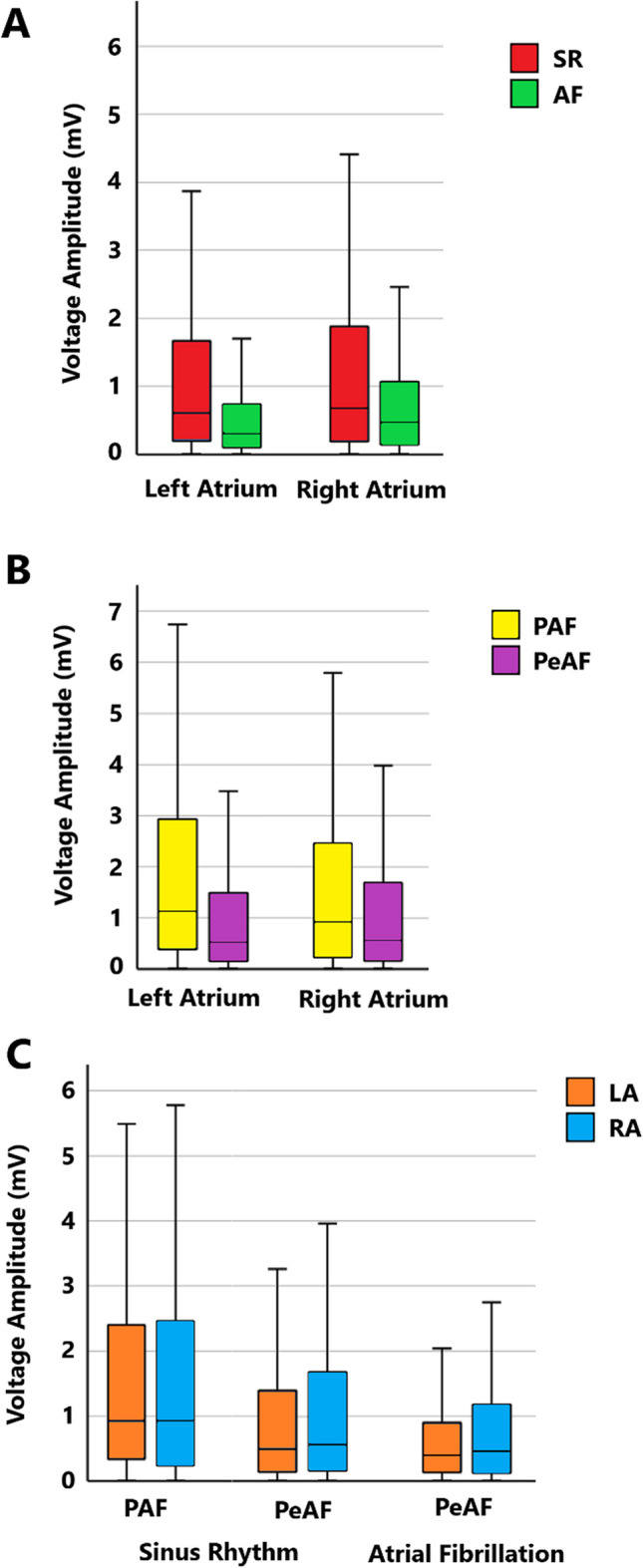
Boxplot comparing electrogram voltage amplitudes. **A** Between sinus rhythm (SR) and atrial fibrillation (AF). **B** Between paroxysmal (PAF) and persistent AF (PeAF). **C** Between left (LA) and right atria (RA). Significant differences (*p* < 0.0005) between groups were seen in all circumstances between rhythms (**A**) and AF types (**B**) (Supplementary Tables 1 and 2). Minimal differences were found between atria on mixed modelling (**C**) (Supplementary Table 3)

**Fig. 3:**
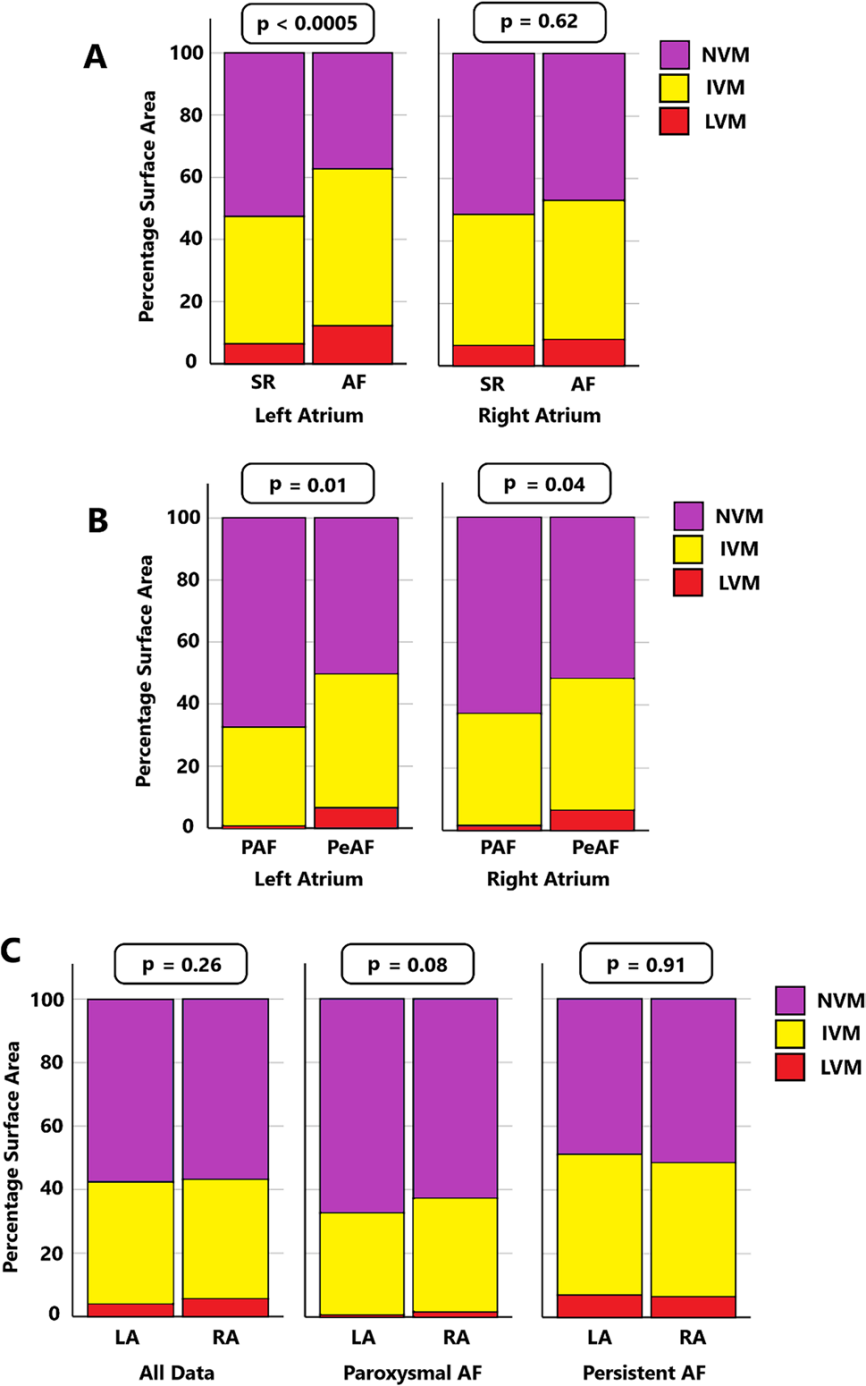
Bar chart comparing the percentage surface area attributed to low voltage (LVM, ≤ 0.05 mV), intermediate voltage (IVM, 0.05–0.5 mV) and normal voltage myocardium (NVM, > 0.5 mV) between **A** sinus rhythm (SR) and atrial fibrillation (AF); **B** paroxysmal (PAF) and persistent AF (PeAF); **C** left (LA) and right atria (RA)

Global median electrogram voltages were significantly higher in patients with PAF over PeAF for both atria, (LA and RA, both *p* < 0.0005, Fig. 2 and Supplementary Table 2). The percentage surface area attributed to different voltage categories were also significantly different between patients with PAF and PeAF in both atria. Both LVM and IVM were higher in PeAF than PAF, with NVM correspondingly lower, (Fig. 3 and Supplementary Table 2).

Typical examples of PAF and PeAF maps are displayed in Supplementary Figs. 2 and 3.

### Left vs right atrium

To compare parameters between atria, the data were divided into PAF and PeAF as this was known to significantly affect electrogram voltages. To allow for comparison of data within patients, generalized linear mixed models were performed.

For patients with PAF, there was minimal difference in SR bipolar voltages between their LA and RA, (Co-efficient comparing LA/RA – 1.06). For patients with PeAF, bipolar voltages were slightly lower in the LA over the RA for both SR and AF, (Co-efficient LA/RA – SR: 0.78; AF 0.70). In all cases, the random factor (patient) was close to the cut-off for statistical significance, suggesting that that there may be patient to patient variation, (Fig. 2 and Supplementary Table 3).

There was no significant difference between atria in individual patients when comparing percentage surface area attributed to LVM/IVM/NVM, (Fig. 3 and Supplementary Table 4).

Typical examples of paired LA and RA maps are displayed in Fig. 1.

## Discussion

### Key findings

This study investigated the differences in quantity and severity of fibrosis between rhythms, AF types and atria using an ultrahigh-density mapping system with voltage amplitude as a surrogate. The key results of the study are the following:

1. Overall, anatomically paired electrograms correlate poorly between SR and AF, and the ability to model each other is low.
2. There is poor agreement between SR and AF electrograms in classification as LVM, IVM or NVM.
3. SR electrograms have a larger amplitude than their corresponding AF counterparts in the same atria—though in a proportion of instances, the converse is also noted
4. The percentage surface area determined as IVM / LVM is greater in AF than SR for the LA, but not the RA.
5. Patients with PeAF have a lower global voltage and more percentage surface area of IVM/LVM than PAF in both atria.
6. Tissue voltages and percentage surface area of IVM / LVM are comparable between atria within a patient.

### Correlating SR and AF electrogram amplitudes

PVI is the cornerstone of AF ablation [10]. However, patients with PeAF and advanced substrate remodelling have increased arrhythmic recurrence post-ablation [11]. One ablation strategy used to improve outcomes is scar guided ablation, which has shown promise in multiple single centre trials [4, 12, 13] and a recent prospective randomised trial [3]. By definition, scar guided ablation is dependent on an accurate substrate map, which in all trials were created in SR. In some patients however, a map in SR is not achievable as the atria are highly susceptible to redeveloping AF, even after DC cardioversion and PVI. In these circumstances, it is necessary to map and ablate during AF. It is therefore desirable to know if low voltage areas seen during AF correspond to those in SR. Unfortunately, our data find the correlations between SR and AF electrogram amplitudes is poor, as is the agreement in classification into LVM, IVM or NVM. Therefore, what is determined to be fibrosis in AF will not be in SR and vice versa, hindering a scar-based ablation strategy.

The poor correlation between paired electrograms is reflective of the chaotic nature of AF. During SR, a constant anatomical source initiates a wave of excitation across fully repolarised tissue with a consistent directionality, resulting in electrograms with minimal variation in amplitude. Conversely in AF, multiple wavelets meander through mixed refractory and repolarised atrial tissue in disorganised re-entrant circuits [14]. This results in a constantly changing wavefront directionality, decreased conduction velocities, inconsistent electrogram amplitudes and fractionation.

The results seen contrast with those of previous studies. Yagishita et al. noted a strong correlation of voltage amplitudes between rhythms of paired bipolar electrograms (Pearson’s *r* = 0.707) [9]. Similarly, Masuda et al. found a moderate correlation (*r* = 0.56), whilst importantly noting the strength of this relationship was dependent on whether SR bipolar electrograms became fractionated during AF, (SR: normal, AF: fractionated, *r* = 0.29; SR and AF: normal, *r* = 0.73) [8]. The weak correlations seen in our study may be explained by high levels of fractionation during AF, particularly reflecting the large surface area of IVM-LVM in our cohort (Mean – LA: 48.3%, RA: 48.5%). Alternatively, different sampling methods, (automated in the current study versus manual in prior work), and catheters used may have affected the results. Ideally, our study would have also been able to classify electrograms by levels of fractionation, but this was impractical to perform manually due to the sheer volume of data obtained.

Clinically, these findings are pertinent, as they suggest that patients are unable to maintain SR for the length of time required to construct a 3D EAM, would be suboptimal candidates for a scar guided strategy. Adoption of such a strategy in this circumstance may result in excess, unnecessary ablation of areas deemed to be scar on an AF voltage map but which are in fact healthy tissue. In these patients, other ablation strategies such as posterior wall isolation may need to be undertaken, or their scar assessed in an alternative manner, for example late gadolinium enhanced magnetic resonance imaging (LGE-MRI)[15].

### SR vs AF electrogram amplitudes and fibrotic surface area

Electrogram amplitudes were found to be overall significantly smaller in AF than SR for both atria, a result consistent with previous studies [9, 16]. As stated above, the electrically chaotic nature of AF results in varying and reduced electrogram amplitudes explaining this result. From a clinical perspective, the greater electrogram amplitudes seen in SR and the lack of electrogram-to-electrogram variation would suggest that it would be a more predictable surrogate for underlying atrial fibrosis than AF. However, whilst this is true generally, a considerable proportion of the paired SR-AF electrograms were higher in AF than SR which in many cases resulted in a healthier tissue categorisation. This may highlight the limitation of direction dependency in bipolar mapping in SR. Although there is the benefit of uniformity in wavefront direction resulting in smaller electrogram amplitude variability, if the alignment of the propagating wavefront is perpendicular to the sampling bipole, the electrogram amplitude could be significantly underestimated. In contrast, the chaotic nature of AF may result in a wavefront propagating towards a bipole in multiple directions in close succession, which could result in a potentially greater reading.

Based on these results, we feel that given the choice, creating a 3D EAM in SR would be a more accurate reflection of atrial tissue health than mapping in AF. However, it should be appreciated that despite ultrahigh-density technology, limitations still exist, and it cannot be considered a ‘gold-standard’.

As histological validation of electrogram voltages with fibrosis has not been studied, one non-invasive method of investigating this relationship uses signal intensity of LGE-MRI. A recent meta-analysis noted 19 of 22 studies found a significant correlation between LGE signal intensity and low voltage areas, however the analysis also highlighted a large heterogeneity between studies, hampering interpretation of the results [17]. Curiously however, in the only study comparing 3D EAMs in both SR and AF to LGE-MRI within the same patient, Quereshi et al. found a significant correlation between LGE signal intensity and bipolar voltages in AF, but not in SR [15]. These results suggest greater understanding of the relationships between LGE-MRI and 3D EAMs; representation of fibrosis is still required.

Interestingly, despite both atria having lower voltages in AF than SR, the RA did not show significantly more scars (IVM/LVM) between rhythms. This may suggest that the fibrillatory waveforms are more organised and less fractionated in the RA compared to the LA.

### Paroxysmal AF vs persistent AF

The pathological progression of AF from paroxysmal to persistent types has been shown to be consistent with increased mural fibrosis demonstrated on MRI [18], 3D EAM studies [2, 4, 19] and autopsy [1]. This is logical as increased fibrosis harbours a greater number of triggers for AF [20] and substrate for enhanced anisotropy and micro re-entry [21]. Our study is consistent with these findings with lower global atrial voltages being found in patients with PeAF than PAF. Logically following from this, an increased surface area of diseased myocardium was also noted in PeAF. Furthermore, by demonstrating decreased tissue voltages in PeAF compared to PAF in the RA, it suggests that it undergoes similar fibrotic changes as the LA with AF progression.

Also of note is the significant quantity of atrium classified as diseased (LVM/IVM) in both PAF (LA: 32.7%; RA: 37.3%) and PeAF (LA: 47.7%, RA: 48.5%), reflecting the widespread nature of the fibrotic pathophysiologic process that characterises AF. These values are higher than comparable studies using the other mapping systems [2, 19], perhaps due to the ultrahigh-density mapping system used in our work or our cohort having an advanced stage of AF.

### Left vs right atrium

Outside of the pulmonary veins, several sites acting as triggers for AF have been documented [2, 22]. In general, non-pulmonary vein trigger sites in the LA are associated with low voltage areas of the LA body (posterior wall, septum). In contrast, in the RA, trigger sites are typically associated with the venous system, (crista terminalis, superior vena cava, coronary sinus) [2, 23]. However, 25% of rotational activity has been documented in the RA [24], whilst electrogram fractionation and localised sources detected by automated algorithms have been shown to be equally distributed across atria [25]. Furthermore, low voltage extensions of the crista terminalis have also been shown to be associated with AF [26], all highlighting that the RA possesses the necessary substrate to maintain AF. In our study, minimal differences in electrogram voltages were seen between atria for PAF but were slightly lower in the LA for PeAF. This could suggest that AF is a progressive bi-atrial fibrotic disease with a minor predominance for the LA. Alternatively, the anatomical structure of the RA with multiple electrically inert structures such as the crista terminalis, the venae cavae and tricuspid valve may reduce the viable pathways for tissue excitation which lend itself to a more organised wavefronts of depolarisation within AF.

Consequently, when performing ablation beyond PVI, mapping the RA may provide significant additional information to guide a scar-based strategy.

### Limitations

Due to the sheer volume of data collected, only electrogram amplitude was considered as a marker of tissue health as an exported value. Ideally other indicators such as electrogram fractionation and their changes between SR and AF would have been explored. Conventional cut-offs of 0.05 mV and 0.5 mV for dense scar and diseased atrial tissue were used; however, these values are not histologically validated. Other higher values suggested by other studies, may produce different results [19, 27]. This study used the Orion mapping catheter; caution should be used when extrapolating of results to other catheters and mapping systems, particularly with different electrode surface areas, inter-electrode spacing and configurations. Contact is known to affect the size of an electrogram collected [28], as the maps were collected by a non-contact force sensing multipolar catheter, some of the variance observed may have been related to differences in contact. In view of the large amount of data collected per map, and the efforts made as far as possible to generate a complete map, we would hope that this limitation would be minimised for this study. Ideally, the relationship between mapped voltages and arrhythmic prognosis would have been investigated. However, the study was not powered to undertake this.

## Conclusions

Ultrahigh-density mapping shows paired electrograms correlate poorly between SR and AF. Anatomically paired SR electrograms are typically (but not always) larger than those in AF. Patients with PeAF have a lower global electrogram voltage than those with PAF. Electrogram voltages are similar between atria within individual patients.

### Supplementary information

Below is the link to the electronic supplementary material.

Supplementary file1 (PDF 511 KB)

## Data Availability

The data underlying the results of this study would be made available upon reasonable request to the corresponding author.
