# Supplementary material for: Comparison of voltages between atria: differences in sinus rhythm and atrial fibrillation
Source: J Interv Card Electrophysiol. 2023 Oct 24;67(3):649–56. doi: 10.1007/s10840-023-01671-0 (PMC11016007; doi:10.1007/s10840-023-01671-0)
Supplement: Supplementary file 1 — Supplementary file1 (PDF 511 KB) [file 10840_2023_1671_MOESM1_ESM.pdf]

Supplementary Materials

Supplementary Table 1: Comparison of Electrogram Voltages and Mapped Surface Area between Sinus Rhythm vs Atrial Fibrillation

**Paired Voltages (mV)**		**Sinus Rhythm**	**Atrial Fibrillation**	**P – Value**
**Left Atrium**		0.61 [0.20 – 1.67]	0.31 [0.10 – 0.74]	All < 0.0005
**Right Atrium**		0.68 [0.19 – 1.88]	0.47 [0.14 – 1.07]	
**Mapped Surface Area (%)**				
**Left Atrium**	LVM	7.3 ± 6.9	12.2 ± 9.5	< 0.0005
	IVM	41.0 ± 12.1	50.5 ± 10.0	
	NVM	51.7 ± 17.2	37.3 ± 16.1	
**Right Atrium**	LVM	6.5 ± 5.3	8.4 ± 8.3	0.62
	IVM	42.0 ± 10.1	44.6 ± 6.9	
	NVM	51.5 ± 13.6	47.0 ± 13.8	

Data is mean ± standard deviation or median [lower quartile – upper quartile]. IVM: Intermediate voltage myocardium; LVM: Low voltage myocardium; mV: Millivolts; NVM: Normal voltage myocardium

Supplementary Table 2: Comparison of Electrogram Voltages and Mapped Surface Area between Paroxysmal and Persistent Atrial Fibrillation

**Global Voltages (mV)**		**Paroxysmal**	**Persistent**	**P – Value**
**Left Atrium**	Bipolar	1.13 [0.39 – 2.93]	0.52 [0.16 – 1.49]	All < 0.0005
**Right Atrium**	Bipolar	0.93 [0.24 – 2.46]	0.57 [0.17 – 1.69]	
**Mapped Surface Area (%)**				
**Left Atrium**	LVM	0.7 ± 0.7	6.6 ± 6.8	0.01
	IVM	32.0 ± 10.6	43.1 ± 11.4	
	NVM	67.3 ± 11.0	50.3 ± 16.4	
**Right Atrium**	LVM	1.6 ± 1.3	6.5 ± 5.3	0.04
	IVM	35.7 ± 10.1	42.0 ± 10.1	
	NVM	62.7 ± 12.1	51.5 ± 13.6	

Data is mean ± standard deviation or median [lower quartile – upper quartile]. Maps were taken in sinus rhythm with proximal coronary sinus pacing. IVM: Intermediate voltage myocardium; LVM: Low voltage myocardium; mV: Millivolts; NVM: Normal voltage myocardium

Supplementary Table 3: Comparisons of Electrogram Voltages between Atria using Generalised Linear Mixed Modelling

Voltages (mV)		Left Atrium	Right Atrium	LA/RA	95% Confidence Interval	Random Factor P-Value
**Paroxysmal**	SR Bipolar	0.93 [0.34 – 2.40]	0.93 [0.24 – 2.46]	1.06	1.05 – 1.07	0.06
**Persistent**	SR Bipolar	0.50 [0.15 – 1.40]	0.57 [0.17 – 1.69]	0.78	0.77 – 0.79	0.02
	AF Bipolar	0.40 [0.14 – 0.90]	0.47 [0.13 – 1.18]	0.70	0.69 – 0.71	0.03

Data is median [lower quartile – upper quartile]. Dependent variable = Electrogram amplitude, Fixed Factor = Left atrium referenced to right atrium; Random factor = Patient. The model used a gamma distribution with logarithmic link function. LA/RA is calculated from converting unstandardised β co-efficient

AF: Atrial Fibrillation; LA: Left Atrium; mV: Millivolts; RA: Right Atrium; SR: Sinus Rhythm.

Supplementary Table 4: Comparison of Mapped Surface Area between Atria

Mapped Surface Area (%)		Left Atrium	Right Atrium	P-Value
**All (n = 17)**	LVM	4.0 ± 6.3	5.6 ± 4.6	0.26
	IVM	38.4 ± 14.0	37.6 ± 10.7	
	NVM	57.5 ± 18.4	56.8 ± 13.8	
**Paroxysmal (n = 8)**	LVM	0.7 ± 0.7	1.6 ± 1.3	0.08
	IVM	32.0 ± 10.6	35.7 ± 10.1	
	NVM	67.3 ± 11.0	62.7 ± 12.1	
**Persistent (n = 9)**	LVM	7.0 ± 7.6	6.5 ± 5.3	0.91
	IVM	44.1 ± 14.7	42.0 ± 10.1	
	NVM	48.9 ± 19.9	51.5 ± 13.6	

Data is mean ± standard deviation. Maps were taken in sinus rhythm with proximal coronary sinus pacing. IVM: Intermediate voltage myocardium; LVM: Low voltage myocardium; mV: Millivolts; NVM: Normal voltage myocardium

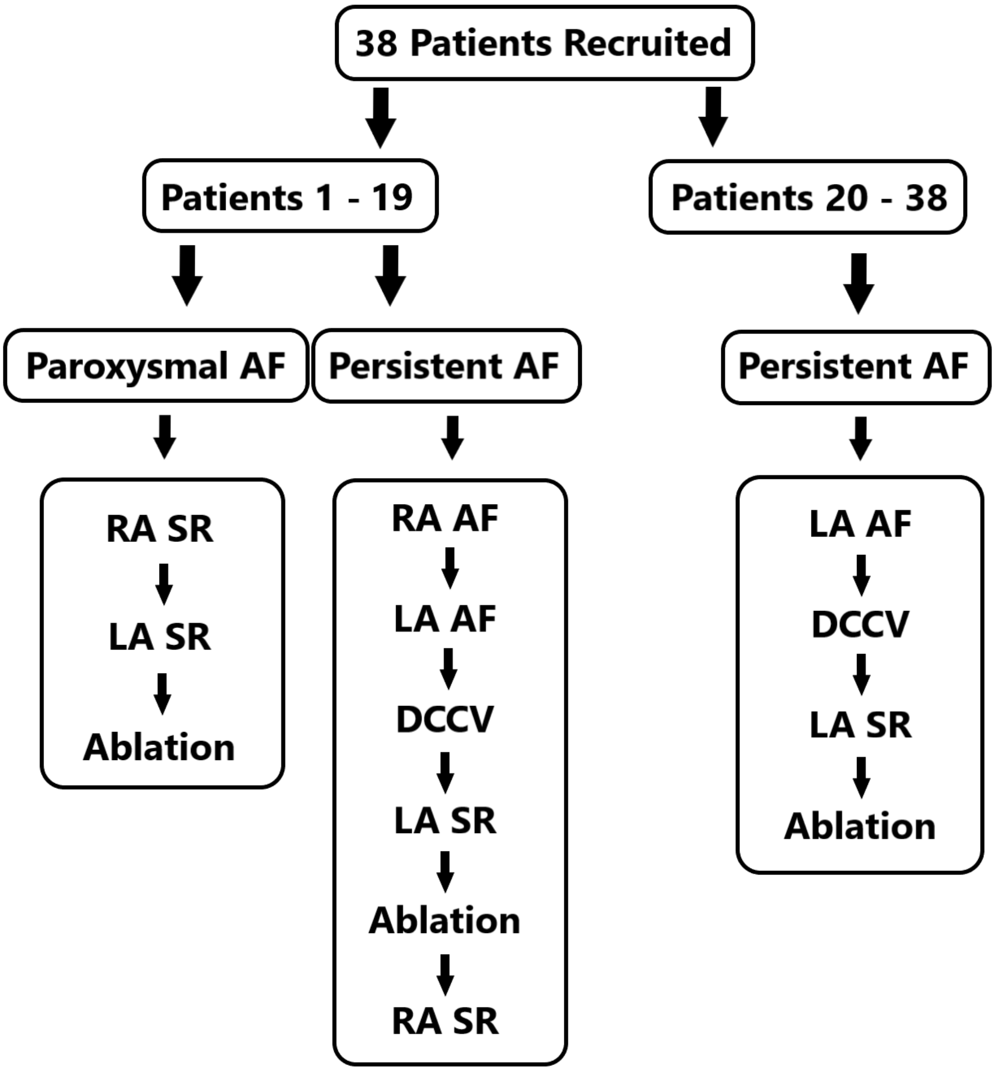

Supplementary Materials Figure 1.

Study mapping protocol. AF = Atrial fibrillation; DCCV = Direct current cardioversion; LA = Left atrium; SR = Sinus rhythm; RA = Right atrium.

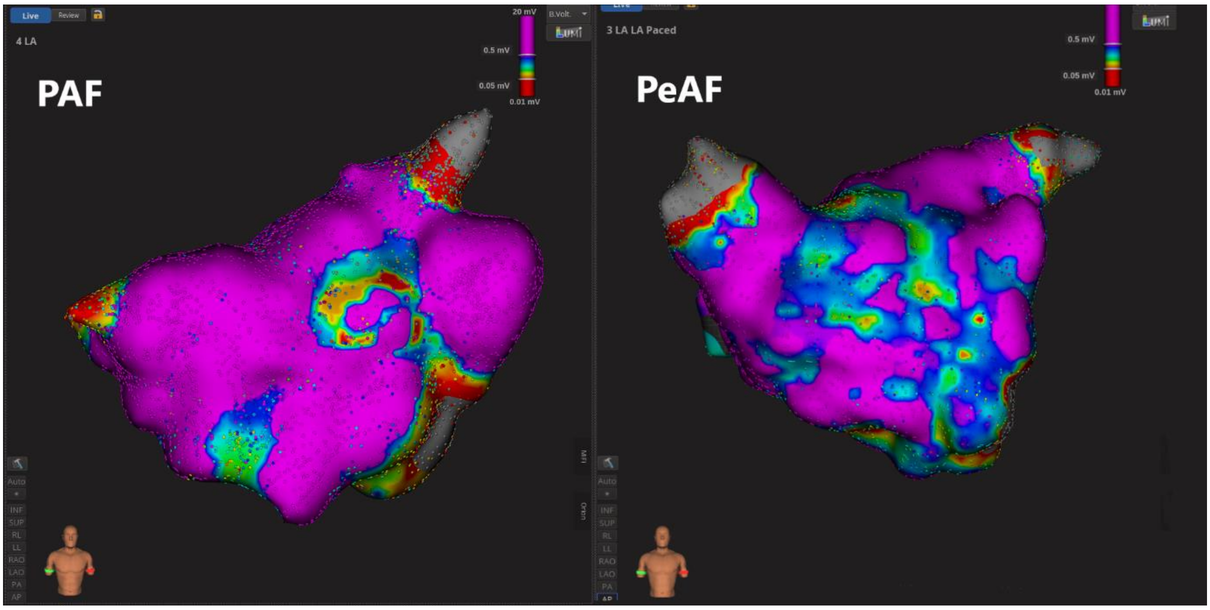

Supplementary Materials Figure 2.

An example of 3D electroanatomical maps of the left atrium in patients with paroxysmal (PAF) and persistent atrial fibrillation (PeAF)

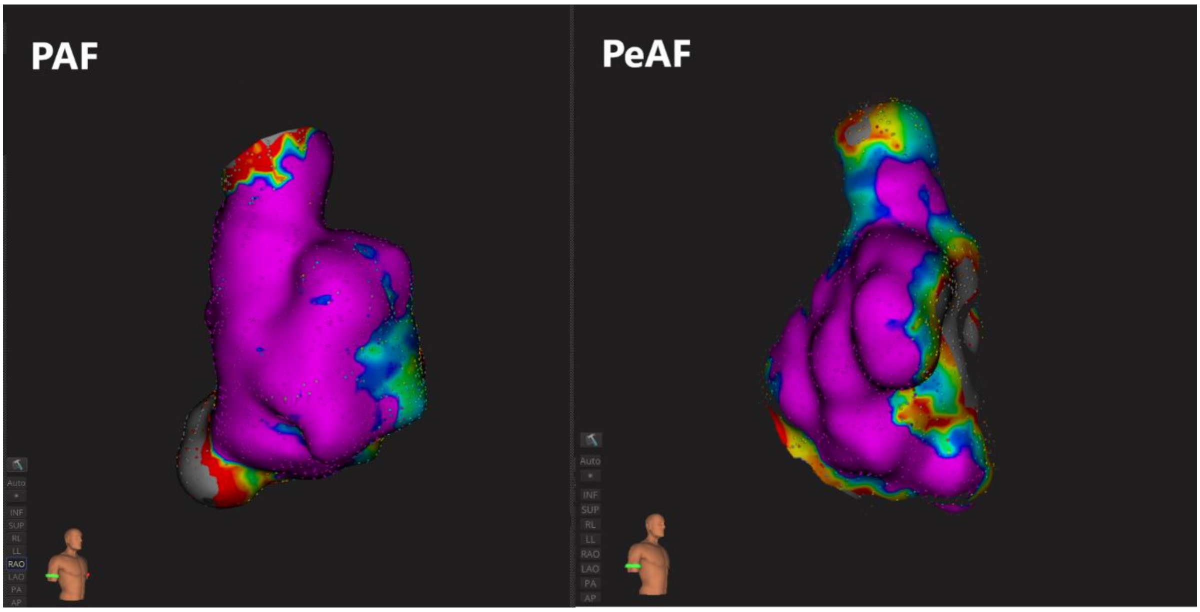

Supplementary Materials Figure 3.

An example of 3D electroanatomical maps of the right atrium in patients with paroxysmal (PAF) and persistent atrial fibrillation (PeAF)
